# Genetic determinants of pig birth weight variability

**DOI:** 10.1186/s12863-015-0309-6

**Published:** 2016-01-27

**Authors:** Xuemin Wang, Xiaolei Liu, Dadong Deng, Mei Yu, Xiaoping Li

**Affiliations:** Key Laboratory of Agricultural Animal Genetics, Breeding, and Reproduction of Ministry of Education, Huazhong Agricultural University, Wuhan, 430070 China; Institute of Animal Science, Jiangsu Academy of Agricultural Science, Nanjing, 210014 China; Institute for Genomic Diversity, Cornell University, Ithaca, NY 14853 USA

**Keywords:** Birth weight variability, GWAS, Glucose and lipid homeostasis, Maternal-fetal lipid transport

## Abstract

**Background:**

Piglet birth weight variability, a trait also known as the within-litter homogeneity of birth weight, reflects the sow’s prolificacy, because it is positively genetically correlated with preweaning mortality but negatively correlated with the mean growth of piglets during sucking. In addition, the maternal additive genetic variance and heritability has been found exist for this trait, thus, reduction in the variability of piglet birth weight to improve the sow prolificacy is possible by selective breeding.

**Results:**

We performed a genome wide association study (GWAS) in 82 sows with extreme standard deviation of birth weights within the first parity to identify significant SNPs, and finally 266 genome-wide significant SNPs (*p* < 0.01) were identified. These SNPs were mainly enriched on chromosome 7, 1, 13, 14, 15 and 18. We further scanned genes of the top 50 SNPs with the lowest p values and found some genes involved in plasma glucose homeostasis (*GLP1R*) and lipid metabolism as well as maternal-fetal lipid transport (*AACS*, *APOB*, *OSBPL10* and *LRP1B*) which may contribute to the birth weight variability trait.

**Conclusions:**

Birth weight variability trait has a low heritability. It is not easy to get significant signal by GWAS using small sample size. Herein, we identified some candidate chromosome regions especially chromosome 7 and suggested five genes which may provide some information for the further study.

**Electronic supplementary material:**

The online version of this article (doi:10.1186/s12863-015-0309-6) contains supplementary material, which is available to authorized users.

## Background

In the past decades, litter size at birth has been considered as the most important index for evaluating sow productivity and great genetic improvement has been successfully obtained for this trait in most of commercial pig breeds [1–4]. However, the preweaning mortality is rather high; thus, a more applicable index for evaluating sow productivity is the total number of alive piglets at weaning produced by a sow per year. Preweaning mortality is influenced by a number of factors, and within-litter variation in birth weight (birth weight variability) has been proved to be an essential factor for piglet survival [5]. Several studies have reported that birth weight variability was positively related to preweaning mortality on the phenotypic scale [5–7]. Recently, several studies have addressed the genetic effect on birth weight variability within-litter. Damgaard et al. [8] analyzed 22,521 piglets born in 2,003 litters by 1,074 Swedish Yorkshire sows and proved the genetic correlation of birth weight variability with proportion of dead piglets and the mean growth of piglets during suckling was 0.25 and −0.31, respectively. Previous studies have also reported the heritability of birth weight variability ranged from 0.08 to 0.12 [8, 9]. Based on the maternal genetic variance and heritability of piglet birth weight variability trait [8], it is possible to improve the genetic progress of this trait by selective breeding. In addition, selection for sows’ capacity to produce homogeneous litters may reduce the piglets’ mortality, improve the mean growth during sucking and obtain more homogeneous litters at weaning, which makes the pig farm “all-in-all-out” strategy possible to get more economic benefits.

The standard deviation of birth weights within one litter can be used to describe the birth weight variability. So far, the genetic architecture of birth weight variability within-litter is still unknown. Genome wide association study (GWAS) using high-density SNP chip such as the Illumina porcine 60K SNP chip has been proved as an efficient tool to identify and map candidate genes for quantitative traits in pigs [10-12]. In this study, we collected 3,305 piglet’s birth weight records from 335 Suzhong sows’ first parities and assigned the sample standard deviation of birth weights within-litter as a phenotypic trait of the sow. Then we performed a GWAS in 82 sows (39 with low variability and 43 with high variability) by using the Illumina porcine 60K SNP chip to identify significant SNPs associated with the birth weight variability at a genome level, then identified the major candidate genes associated with this trait. The filtered SNP loci may be used as a preliminary foundation for further selective breeding.

## Results and discussion

### Genome-wide significant SNPs from the association studies

**Fig. 1:**
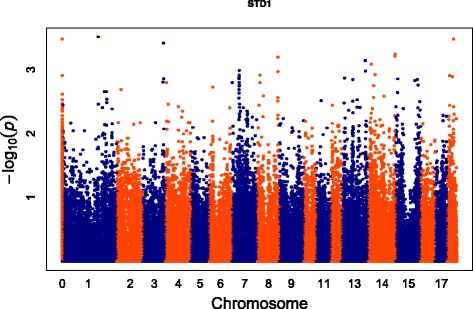
Manhattan-Plot for association of SNP loci with birth weight variability. The X axis indicates in different colors from left to right, SNP locations from chromosomes 1–18 (chromosome location for unmapped SNPs was represented by 0), using Sus scrofa genome build10.2. The Y axis represents the minus log of the P-value for each SNP

**Fig. 2:**
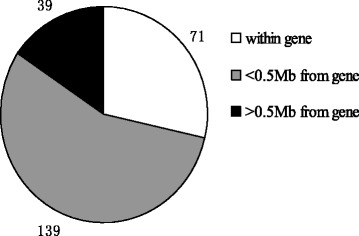
The genomic distribution of the 249 significant SNPs (*p* < 0.05) associated with piglet birth weight variability

Totally, 53,693 SNPs with genotypes in 82 individuals were used for association analyses after data filtering. The number of genome-wide significant SNPs were 1916 and 266 at α level 0.05 and 0.01, respectively. For the 266 significant SNPs, 17 SNPs have not been mapped to any chromosome, and the other 249 SNPs were mainly enriched on chromosome 1, 7, 13, 14, 15 and 18 (Fig. 1). We further scanned genes of the 249 SNPs located in, and found 71 SNPs were located within 60 annotated genes, 139 SNPs in region of 0.5 Mb away from the nearest genes and no genes had been found in region of 1 Mb for the rest of 39 SNPs (Fig. 2). The detailed information for the top 50 SNPs with the lowest *p* values is illustrated in Table 1.

**Table 1:** The annotated genes between 500 kb downstream and 500 kb upstream of the 50 SNPs with the lowest *p* value from the GWAS

No	SNP name	Pig chromosome	Position (Mb)	*P* value	Adjacent genes^a^ (±0.5 Mb)	Distance^b^ (bp)
1	ALGA0007307	1	206.50	0.0003	*PELI2,* ***TMEM260,*** *OTX2, EXOC5*	***within***
2	DRGA0004275	3	129.20	0.0004	***FAM49A***	***within***
3	ALGA0083116	14	149.00	0.0006	*FOXI2, NPS,* ***PTPRE,*** *MKI67*	***within***
4	ALGA0083057	14	147.76	0.0006	***FAM196A***	488070
5	ASGA0040051	8	139.04	0.0006	*MMRN1*, *SNCA,* ***GPRIN3***	293849
6	MARC0115245	13	188.57	0.0007	NA^c^	
7	ASGA0061743	14	16.41	0.0008	*GATA4,NEIL2,FDFT1, CTSB,* ***DEFB134,*** *ADAM29*	−44520
8	DIAS0000130	7	39.09	0.0010	*ZFAND3,* ***BTBD9,*** *GLO1, DNAH8*	***within***
9	DRGA0013238	13	188.95	0.0010	***LIPI***	380204
10	DRGA0008884	8	139.01	0.0011	*MMRN1* **,** *SNCA,* ***GPRIN3***	317059
11	H3GA0039777	14	37.65	0.0012	*NOS1, FBXO21* ***, TESC,*** *FBXW8, RNFT2*	***within***
12	ALGA0114335	8	17.21	0.0012	*ADGRA3,* ***GBA3***	***within***
13	ALGA0040467	7	38.40	0.0012	*CCDC167,* ***MDGA1*** *,ZFAND3*	−227337
14	ALGA0040474	7	38.89	0.0012	*CPNE5, PPIL1, PI16, MTCH1, FGD2, PIM1, TMEM217, TBC1D22B, RNF8,* ***ZFAND3*** *, CCDC167, MDGA1*	−142259
15	ASGA0083383	18	17.18	0.0012	***CHCHD3***	***within***
16	ALGA0097813	18	32.55	0.0013	***TFEC***	−338821
17	H3GA0020922	7	38.06	0.0013	*FGD2, PIM1, TMEM217, TBC1D22B, RNF8,* ***CCDC167,*** *MDGA1*	−85741
18	ALGA0040434	7	38.08	0.0013	*FGD2, PIM1, TMEM217, TBC1D22B, RNF8,* ***CCDC167,*** *MDGA1*	−106358
19	ALGA0109619	13	20.09	0.0013	*STT3B,* ***OSBPL10,*** *CMTM6, DYNC1LI1, CMTM7, CMTM* ***8***	***within***
20	MARC0090396	3	130.65	0.0014	**ENSSSCG00000008620**	***within***
21	H3GA0019379	7	37.84	0.0014	*PPIL1, PI16, MTCH1, FGD2, PIM1, TMEM217,* ***TBC1D22B,*** *RNF8, ZFAND3, CCDC167, MDGA1*	−93575
22	ASGA0068602	15	12.98	0.0014	***LRP1B***	−50827
23	ALGA0070915	13	81.34	0.0014	*CHST13, ACPP, DNAJC13,* ***ACAD11,*** *UBA5*	***within***
24	DRGA0015381	15	121.31	0.0015	*IN080D,NDUFS1, EEF1B2, GPR1, ZDBF2,* ***ADAM23,*** *FASTKD2, MDH1B, CPO*	***within***
25	ASGA0016323	3	124.90	0.0016	***APOB***	326494
26	MARC0061348	7	38.21	0.0016	*TMEM217, TBC1D22B, RNF8, CCDC167,* ***MDGA1***	−98887
27	DRGA0007508	7	38.82	0.0016	***ZFAND3,*** *BTBD9, GLO1, DNAH8*	−73453
28	MARC0084509	4	5.59	0.0016	NA	
29	ALGA0037853	7	0.43	0.0016	***CCDC167,*** *FOXQ1, FOXF2, GMDS,*	20912
30	ASGA0037952	8	17.25	0.0016	***ADGRA3***	−312721
31	ALGA0087652	15	140.88	0.0017	***NYAP2***	−349642
32	ALGA0040570	7	39.63	0.0018	*GLO1,DNAH8,* ***GLP1R,*** *KCNK5,KCNK17,KIF6*	***within***
33	ASGA0062412	14	29.48	0.0018	***TMEM132B,*** *AACS, BRI3BP, DHX37*	***within***
34	ASGA0032735	7	38.46	0.0018	*CCDC167,* ***MDGA1*** *, ZFAND3*	−316254
35	ALGA0040468	7	38.54	0.0018	*CCDC167,* ***MDGA1*** *, ZFAND3*	202512
36	ASGA0027748	6	18.83	0.0019	*CNOT1,* ***GOT2***	−323698
37	ALGA0012631	2	25.98	0.0020	NA	
38	MARC0008120	7	37.81	0.0020	*CPNE5,PPIL1,PI16,MTCH1,PTGDS, PIM1, TMEM217,* ***TBC1D22B,*** *RNF8, CCDC167, MDGA1*	−69290
39	DRGA0002077	1	254.15	0.0022	***RORB***	461748
40	BGIS0006392	1	244.49	0.0022	***KCNV2***	473499
41	ALGA0083738	15	0.26	0.0023	***NMI***	−227794
42	ASGA0032683	7	37.95	0.0023	*PPIL1,PI16,MTCH1,PTGDS,PIM1, TMEM217, TBC1D22B, RNF8,* ***CCDC167,*** *MDGA1*	−45021
43	ALGA0040427	7	37.97	0.0023	*PPIL1,PI16,MTCH1,PTGDS,PIM1, TMEM217, TBC1D22B, RNF8,* ***CCDC167,*** *MDGA1*	−62182
44	DIAS0003266	13	38.47	0.0024	*GNL3,GLT8D1, SPCS1, NEK4, ITIH3, ITIH4, SFMBT1, PRKCD, TKT,* ***DCP1A***	***within***
45	ALGA0106090	15	2.84	0.0024	*LYPD6B,* ***KIF5C***	***within***
46	ASGA0038720	8	40.37	0.0026	*SLAIN2,* ***SLC10A4*** *, ZAR1*	39595
47	SIRI0000172	14	87.58	0.0026	NA	
48	ALGA0076580	14	29.46	0.0027	***TMEM132B,*** *AACS, BRI3BP, DHX37*	***within***
49	ALGA0049776	8	138.91	0.0027	*MMRN1,* ***SNCA*** *, GPRIN3*	−275407
50	ASGA0032655	7	37.40	0.0028	*PNPLA1,ETV7,STK38,SRSF3,RAB44,* ***CPNE5,*** *PPIL1,PI16,MTCH1,PTGDS,PIM1, TMEM217,TBC1D22B, RNF8*	***within***

^a^gene with black bold is the nearest gene from the SNP. ^b^Positive value denotes the gene located downstream of the SNP, negative value denotes the gene located upstream of the SNP. ^c^no gene has been identified in this region

### Genes associated with glucose and lipid metabolism and transport

Pregnancy is a critical period for both the mother and the fetus, and the maternal factors can affect fetal growth and pregnancy outcomes. In order to sustain appropriate fetal development, the mother must provide nutrients such as glucose, amino acids and lipids to the fetus across the placenta [13]. And therefore, genes affecting the maternal nutrient ingestion, energy metabolism and maternal-fetal nutrient transport may affect the placental development as well as fetal growth and finally result in the neonatal birth weight variation. In this study, we only focused on the 50 most significant SNPs listed in Table 2 for candidate genes scanning. Among the 50 SNPs, 17 SNPs were located within annotated genes. Particularly worth mentioning is that, 19 SNPs were mapped on SSC7 spanning from 37.4 Mb to 39.6 Mb. In this region, we explored 27 annotated genes including one gene *GLP1R* (*p* = 0.0018) in regulating plasma glucose levels. From the 50 SNPs we also explored several genes involved in lipid metabolism such as *AACS* on SSC14 (*p* = 0.0018), and lipid transport related proteins including *APOB* on SSC3 (*p* = 0.0016), *OSBPL10* on SSC13 (*p* = 0.0013), and *LRP1B* on SSC15 (*p* = 0.0014).

**Table 2:** The expression of five candidate genes in porcine placenta and endometrium analyzed by using public microarray and RNA-seq data

Gene name	Placenta	Endometrium
AACS	Microarray, +, Meishan and white composite [59];	Microarray, +, Meishan and Yorkshire [60];
	RNA-seq, RPKM = 11.78, Duroc and wild boar [61]	RNA-seq, RPKM = 11.92 [62]
OSBPL10	Microarray, +, Meishan and white composite [59];	Microarray, +, Meishan and Yorkshire [60];
	RNA-seq, RPKM = 9.42, Duroc and wild boar [61]	RNA-seq, RPKM = 8.94 [62]
APOB	Microarray, +, Meishan and white composite [59];	Microarray, +, Meishan and Yorkshire [60];
		RNA-seq, RPKM = 2.04 [62]
LRP1B		RNA-seq, RPKM = 0.12 [62]
GLP1R		RNA-seq, RPKM = 0.11 [62]

Glucose is the primary energy substrate essential for the fetal growth and development. However, fetus generates minimal glucose by itself, and most of the glucose is transported from maternal circulation through the glucose transporters [14]. During the transport, the maternal glucose should be taken up by placenta firstly, then entries into the fetal circulation across two layers of cells [15–18]. The *GLP1R* gene encodes glucagon-like peptide 1 (GLP1) receptor, which specifically binds with GLP1 to mediate its biological actions [19–21]. In mammals, stimulation of the *GLP1R* in the pancreatic β cells results in a rise of insulin secretion and lowers plasma glucose levels [22, 23]. The maternal plasma glucose levels dramatically affect the fetal growth, because glucose is the nutrient that crosses the placenta in the greatest quantities by facilitated diffusion along a concentration gradient [24]. A number of studies have demonstrated that abnormal maternal plasma glucose level such as hyperglycemia (hypoglycemia) is associated with fetal overgrowth (restriction) during pregnancy [25–30].

Except for glucose, lipids such as triglycerides (TGs) and cholesterol serve many critical roles in fetal growth [31]. The *AACS* gene encodes acetoacetyl-CoA synthetase, which is an acetoacetate-specific ligase [32]. Acetoacetate is the ketone body substrate for lipid biosynthesis which can be converted into acetoacetyl-CoA by AACS then subsequently used for the synthesis of cholesterol or fatty acid. Knock down of AACS in mouse significantly reduced the total blood cholesterol [33], which suggested AACS may play an important role in plasma cholesterol homeostasis.

Cholesterol is a kind of lipids that plays important roles in fetal development, as it is an essential component of cell membranes, a precursor for steroid hormones and is also essential for activation of various signaling pathways [34, 35]. Although most of the fetus’s cholesterol is synthesized by the fetus itself, more and more evidence suggested that during the first weeks of life, the fetus largely depends on maternal cholesterol as its cholesterol source [36]. The maternal cholesterol is initially taken up by the placenta, and then transported to the fetus by the cholesterol-carrying lipoproteins [37, 38]. The apolipoprotein B (apoB)-containing lipoproteins is an efficient system for delivery of lipids because these lipoproteins contain large amounts of cholesterol, TGs and essential lipids [39]. ApoB (encoded by the *APOB* gene) is the principal protein component of plasma very low density lipoproteins (VLDL) and low density lipoproteins (LDL), and several genome-wide association studies in pig populations have revealed the *APOB* gene was associated with the serum total cholesterol (TC) and LDL cholesterol (LDL-C) levels [40–42]. ApoB is also an essential component for the assembly and secretion of competent apoB-containing lipoproteins [43, 44]. Human and rat placenta can synthesize and secrete apoB [45–47], and a sharp increase in rat placental apoB mRNA during the last 48 h of pregnancy has been reported by Demmer et al. [48]. Mouse yolk sac also secretes apoB, and embryos lacking apoB can not export lipoproteins from yolk sac endoderm cells and die with severe neuro-developmental abnormalities during mid-gestation [49, 50]. All these studies suggest a specific role for the *APOB* gene in maternal-fetal lipid transport.

In mammals, oxysterols are oxygenated forms of cholesterol. Oxysterol-binding protein (OSBP) and its homologs OSBP-related (ORP) or OSBP-like (OSBPL) proteins constitute a conserved family of lipid binding/transfer proteins (LTP), which can accommodate cholesterol, oxysterols and other steroids. The OSBPL10 (also known as ORP10) is a member of the LTP family and has the capacity to bind cholesterol and several acidic phospholipids [51]. Association studies revealed polymorphisms in the *OSBPL10* gene displayed linkage and association with the extreme upper end serum triacylglycerol (TAG) and LDL-C levels in dyslipidaemic subjects [52, 53]. Functional studies have also demonstrated the *OSBPL10* gene negatively regulates hepatocellular VLDL biosynthesis and suppresses apoB-containing lipoproteins secretion [51].

**Fig. 3:**
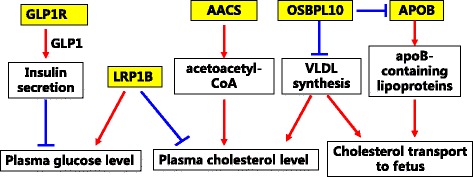
Summary of the five candidate genes involved in glucose and lipid homeostasis as well as maternal-fetal lipid transport pathways

Finally, *LRP1B* gene encodes LDL receptor-related protein 1B and mediates cellular cholesterol uptake [54]. Dietrich et al. [55] reported that knockout of Lrp1b in mice results in early embryonic lethality. Association analysis identified LRP1B as a determinant of rat cholesterol concentrations in LDL, and a significant association with child body mass index (BMI) in human [56, 57]. Furthermore, recent studies suggested the *LRP1B* gene was also involved in glucose homeostasis. Polymorphism of this gene was associated with insulin resistance and in normoglycemic women the maternal glucose levels were associated with DNA methylation changes at *LRP1B* gene loci in the placenta and cord blood [57, 58]. We summarized the above five candidate genes (*GLP1R*, *AACS*, *APOB*, *OSBPL10* and *LRP1B*) involved in glucose and lipid homeostasis as well as maternal-fetal lipid transport pathways in Fig. 3.

### Expression of candidate genes in porcine placenta and endometrium tissues

Genes expressed in placenta or endometrium may play functional roles for fetal development, and the public RNA-seq data give us a good opportunity to check gene expression in specific tissues. We checked the above five candidate genes (*GLP1R*, *AACS, APOB*, *OSBPL10* and *LRP1B*) in porcine placenta and endometrium tissues by using the public RNA-seq data or microarray data, and the results were summarized in Table 2. We found the *AACS* and *OSBPL10* gene had relatively high expression both in porcine placenta and endometrium at different gestational stages (day 25, 45, 65, 85, 105 and 113) [59–62]. However, the other two genes *GLP1R* and *LRP1B* had no expression in these two tissues from the above data. The *APOB* gene had very low expression in porcine endometrium during early (gestational day 15) and mid-gestation (gestational days 26 and 50) [60, 62]. Based on the Yorkshire endometrium RNA-seq deep sequencing results (Size = 10Gb, unpublished data from our group), the *AACS* gene was highly expressed in the endometrium during early embryo implantation (RPKM > 250) and the expression of *APOB* gene was sharply increased at gestational day 15 (RPKM = 16.72) compared with day 12 (RPKM = 1.60).

Interestingly, we found half of the 27 genes on SSC7 listed in Table 1 expressed in porcine placenta (gestational day 113) and endometrium (gestational day 25) including four extremely high expression genes (*ZFAND3*, *FOXQ1*, *GMDS* and *MTCH1*) [50, 51]. *ZFAND3* gene encodes Zing finger AN1-type domain 3 protein which is originally isolated from the mouse testis [63] and expression assay suggested this gene is involved in spermatogenesis [64]. Recently, association studies identified *ZFAND3* as a susceptible gene to type 2 diabetes in several human populations [65, 66], which suggested this gene may be involved in plasma glucose homeostasis. The high expression of *ZFAND3* gene in porcine placenta and endometrium possibly imply its functional role for embryo (or fetus) development.

## Conclusions

Birth weight variability is an economic trait with low heritability. In this study, we performed a GWAS in 82 sows with extreme phenotypic records and identified 266 significant associated SNPs (*p* < 0.01). For the top 50 significant SNPs, we further scanned the genes within 1 Mb region and finally suggested candidate genes involved in plasma glucose homeostasis (*GLP1R*) and lipid metabolism as well as maternal-fetal lipid transport (*AACS, APOB*, *OSBPL10* and *LRP1B*) which may contribute to the current trait we focused on. But, further association analysis in bigger sample size and function studies need be carried out to confirm our present conclusion.

## Methods

### Pig population and phenotype measurement

The pigs we studied were coming from Suzhong pig seed farm of Jiangsu Academy of Agricultural Institute. We collected the reproductive information from 335 sows, including the total number born (TNB), number born alive (NBA) and the birth weight (BW) records from total of 3,305 first parity’s piglets. Because the farm only had the birth weight of born alive offspring’s records, in this case the sample standard deviation (SD) of born alive birth weights within one litter was described as a phenotype to assess the piglet birth weight variability for each sow.

### Genotyping and quality control

A total of 82 sows with extreme SD were genotyped for further association studying, and they were divided into low (group 1, *n* = 39) and high (group 2, *n* = 43) SD groups, with the mean SD 0.08 (from 0.04 to 0.12) and 0.21 (from 0.12 to 0.48), respectively. The TNB, NBA and SD information of these sows is summarized in Additional file 1. It is worth mentioning that the TNB is more than four for all the 82 studied sows in order to reduce the effect of litter size. 5 ml blood samples were collected from each sow for genomic DNA isolation using a standard phenol/chloroform method. All DNA samples were qualified with a ratio of A260/280 between 1.80 and 2.0 and standardized into a final concentration of 200 ng/μL. Then, 2 μg DNA sample from each of these sows were genotyped using the Porcine SNP60 Beadchips (Illumina, USA) following the manufacturer’s protocol. Quality control was carried out using PLINK (version 1.07) [67] and executed SNPs with call rate < 80 %, Gentrain score < 40 %, minor allele frequency (MAF) < 0.01, and severely departed from hardy weinberg equilibrium (HWE) (*P*-value < 0.0001).

### Genome-wide association analyses

In this study, compressed mixed linear model (CMLM) from the Genome Association and Prediction Integrated Tool (GAPIT) program package [68] was used for whole genome association analyses. The CMLM statistical model we used was described as following:

y = Xα + Pβ + Kμ + e. Where y is the vector of phenotype, X is a matrix of SNP genotypes, p is a matrix of PC (principle components) for population structure, K is a kinship matrix. Xα and Pβ are regarded as fixed effects, where Pβ is used as a covariate to address the spurious associations that arise from population structure, and Kμ and e are regarded as random effects.

### Gene search and functional annotation

Gene searches were carried out in 0.5 Mb sequence upstream and downstream of the significant associated SNPs with the top 50 lowest p value using the Sus scrofa 10.3 genome build. If no genes were identified in the gene-poor regions, then the genes upstream and downstream of the region were considered to possibly represent the locus. Functional annotation clustering was performed for all the identified genes using DAVID software (http://david.abcc.ncifcrf.gov), and the gene enrichment clusters related to reproductive functions and reproductive tissues were taken into consideration.

## Additional file

Additional file 1:
**Phenotype records of the 82 sows used for GWAS.** (XLS 2366 kb)

## Abbreviations

GWAS: genome wide association study
SD: standard deviation
SNPs: single nucleotide polymorphisms
MAF: minor allele frequency
HWE: hardy weinberg equilibrium
Mb: mega base
SSC: sus scrofa chromosome
CMLM: compressed mixed linear model
GAPIT: Genome Association and Prediction Integrated Tool
DAVID: The Database for Annotation, Visualization and Integrated Discovery
TGs: triglycerides
VLDL: very low density lipoproteins
LDL: low density lipoproteins
TC: total cholesterol
LDL-C: low density lipoprotein cholesterol
TAG: triacylglycerol
GLP1R: glucagon-like peptide 1 receptor
AACS: acetoacetyl-CoA synthetase
APOB: apolipoprotein B
OSBPL10: Oxysterol-binding protein like 10
LRP1B: LDL receptor-related protein 1B
BMI: body mass index
RPKM: Reads Per Kilobase of exon model per Million mapped reads
TNB: total number born
NBA: number born alive
BW: birth weight
